# Real‐world utility of next‐generation sequencing for targeted gene analysis and its application to treatment in lung adenocarcinoma

**DOI:** 10.1002/cam4.3874

**Published:** 2021-05-07

**Authors:** Jwa Hoon Kim, Shinkyo Yoon, Dae Ho Lee, Se Jin Jang, Sung‐Min Chun, Sang‐We Kim

**Affiliations:** ^1^ Department of Oncology Asan Medical Center University of Ulsan College of Medicine Seoul Republic of Korea; ^2^ Division of Oncology/Hematology Department of Internal Medicine Korea University Anam Hospital Korea University College of Medicine Seoul Republic of Korea; ^3^ Department of Pathology Asan Medical Center University of Ulsan College of Medicine Seoul Republic of Korea

**Keywords:** lung adenocarcinoma, next‐generation sequencing, survival, targeted therapy

## Abstract

**Purpose:**

This study investigated the clinical utility of next‐generation sequencing (NGS) for detection of genetic alterations and its implications on treatment of lung adenocarcinoma in real‐world practice.

**Patients and Methods:**

Data were reviewed for 391 patients with lung adenocarcinoma who underwent NGS between March 2017 and October 2018. Formalin‐fixed, paraffin‐embedded archival samples were used for performing NGS targeting 382 genes, including all exons of 199 genes, 184 hotspots, and the partial introns of 8 genes often rearranged in cancer. Survival analysis was performed for stage IV disease.

**Results:**

Among the 391 patients, at least one actionable mutation was identified in 294 patients (75.2%). The most commonly mutated gene was *EGFR* (*n* = 130, 33.2%), involving *EGFR* exon 19 deletion (*n* = 48, 12.3%), L858R (*n* = 47, 12%), and others (*n* = 35, 9%), followed by *KRAS* (*n* = 48, 12.3%), *ALK* (*n* = 40, 10.2%), *RET* (6%), *MET* (3%), *ROS*‐*1* (3%), and *BRAF* (2%) mutations. *TP53* (46.9%) and *CDKN2A* (12.6%) mutations were common co‐mutations in patients with AMs. With a median follow‐up duration of 16.8 months, median overall survival was 36.8 months in patients with stage IV disease. Patients treated with the corresponding targeted therapy for AMs based on NGS reports lived significantly longer than those not treated with such therapy (*p* < 0.001). After multivariate analysis, targeted therapy for AM was a significantly favorable factor for survival (AM without targeted therapy vs. AM with targeted therapy, hazard ratio 2.58, 95% confidence interval 1.57–4.25; *p* < 0.001).

**Conclusion:**

This study revealed that AMs could be comparably detected using NGS. Based on these NGS results, a suitable targeted therapy can be selected, which may improve survival in patients with lung adenocarcinoma. This NGS‐based approach is useful in real‐world practice to provide guidance when selecting targeted therapy.

## Graphical abstract

This study showed clinical utility of next‐generation sequencing (NGS) in real‐world practice showing the landscape of actionable genetic alterations in 391 patients with lung adenocarcinoma. The NGS test can be useful in identifying even rare ones as well as common mutations, which can allow patients receive targeted therapy with better survival. This NGS‐based approach can be useful in real‐world practice to provide guidance when selecting targeted therapy.

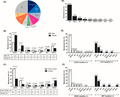

## 1 INTRODUCTION

Lung cancer is the leading cause of cancer death globally. It is also the third most common cancer, and its 5‐year survival rate is only 28.2% in South Korea.^1^ Since the introduction of targeted therapy for lung cancer a decade ago,^2^, ^3^ its prognosis has improved. The response rate with targeted therapy in molecularly selected patients has been reported to be up to 70% compared with 20%–30% in unselected patients with conventional chemotherapy.^4^ Consequently, tyrosine kinase inhibitors targeting epidermal growth factor receptor (*EGFR*) and anaplastic lymphoma kinase (*ALK*) have become the standard treatment for patients with metastatic *EGFR*‐ or *ALK*‐mutated lung adenocarcinoma, and these tests are performed in routine practice.^5^ Recent discoveries of *ROS*‐*1* and *RET* rearrangement have led to the development of novel targeted agents, and *BRAF*, *MET*, and *NTRK* mutations have also emerged as targets in the treatment of lung cancer.^4^ Comprehensive profiling of genetic alterations to search target biomarkers is now being actively encouraged for patients with lung cancer.

The need to genotype tumors in the era of personalized therapy has been raised not only for lung cancer but also for other cancer types. Along with technological progress, next‐generation sequencing (NGS) has certain strengths, including the easy identification of multiple genetic alterations simultaneously, which saves time compared with sequential conventional tests, such as single‐targeting polymerase chain reaction (PCR) and immunohistochemistry; moreover, NGS exhibits high sensitivity. In this regard, several NGS‐based projects^6^, ^7^, ^8^ have been performed worldwide to identify a wide range of clinically relevant genetic alterations within multiple tumor types.

However, the published data have focused on Caucasian patients and data on Asian patients obtained using NGS are lacking. Its clinical usefulness is also unclear in real‐world practice. Given that there are genetic variations between different ethnic groups and different routine practice circumstances in clinical trials, it is worth investigating the genetic alterations of Korean patients in real‐world practice. Furthermore, since the National Health Insurance Service of Korea broadened its insurance coverage, NGS has been actively encouraged in actual clinical practice, and recently, NGS data on various tumor types have been published in Korea.^7^ Against this background, this study investigated the clinical utility of NGS for the detection of genetic alterations and its implications on treatment in patients with lung adenocarcinoma.

## 2 MATERIALS AND METHODS

### 2.1 Patients and data collection

A total of 391 patients with lung adenocarcinoma who were treated at Asan Medical Center (AMC) (Seoul, South Korea) between March 2017 and October 2018 and underwent NGS at the physician's discretion regardless of undergoing prior conventional single‐targeting PCR for *EGFR* mutation were included in this study. Baseline characteristics collected from the database included age, sex, Eastern Cooperative Oncology Group performance status (ECOG PS), smoking history, history of anticancer treatment, survival outcome, and NGS reports. Never smokers were defined as those with a lifetime smoking dose of <100 cigarettes, and current smokers or ex‐smokers were defined as those with a lifetime smoking dose of ≥100 cigarettes and who currently smoke or have quit smoking. Actionable mutations from the NGS reports were defined as available or potential targets for anti‐cancer treatment and included *EGFR*, *ALK*, *ROS*‐*1*, *KRAS*, *MET*, *RET*, *BRAF*, *ERBB2*, and *PIK3CA* or *PTEN* mutations. Co‐mutation was also identified from the NGS reports. This study was approved by the AMC Institutional Review Board.

### 2.2 DNA extraction

After review of the matched hematoxylin/eosin‐stained slides from each formalin‐fixed, paraffin‐embedded (FFPE) tissue section, 6‐μm‐thick slices from each specimen were used for the extraction of genomic DNA, depending on the sample size and tumor cellularity. After treatment with xylene and ethanol for de‐paraffinization, genomic DNA was isolated using the NEXprep FFPE Tissue kit (#NexK‐9000; Geneslabs), in accordance with the manufacturer's protocol. Briefly, tissue pellets were lysed completely by incubation with proteinase K in lysis buffer overnight at 56°C, followed by additional incubation for 3 min with magnetic beads and Solution A at 37°C. After incubation for 5 min on a magnetic stand, the supernatants were removed and washed three times with ethanol. After air‐drying the beads for 5 min, DNA was eluted in 50 μl of nuclease‐free water and quantified using a Qubit™ dsDNA HS Assay kit (Thermo Fisher Scientific).

### 2.3 Targeted next‐generation sequencing

Targeted NGS was performed using the MiSeq platform (Illumina) with OncoPanel AMC version 3 (OP_AMCv3) targeting a total of 382 genes, including the entire exons of 199 genes, 184 hotspots, and the partial introns of 8 genes often rearranged in cancer (Table S1). Two hundred nanograms of genomic DNA was fragmented by sonication (Covaris Inc.) to an average size of 250 bp, followed by size selection using Agencourt AMPure XP beads (Beckman Coulter). A DNA library was prepared by sequential reactions of end repair, A‐tailing, and ligation with a TruSeq adaptor, using a SureSelectXT Reagent kit (Agilent Technologies). Each library was addressed with sample‐specific barcodes of 6 bp and quantified using Qubit. Eight libraries were pooled to a total of 750 ng for hybrid capture using an Agilent SureSelectXT custom kit (OP_AMCv3 RNA bait; Agilent Technologies). The concentration of the enriched target was measured by quantitative PCR (Kapa Biosystems), and the sample was loaded on the MiSeq platform or subjected to paired‐end sequencing.

### 2.4 Bioinformatics analysis

Sequenced reads were aligned to the human reference genome (NCBI build 37) with the Burrows‐Wheeler Aligner (0.5.9)^9^ with the default options. De‐multiplexing was performed with MarkDuplicates of the Picard package to remove PCR duplicates (available at http://broadinstitute.github.io/picard). De‐duplicated reads were re‐aligned at known indel positions with the GATK IndelRealigner tool.^10^ The base quality was recalibrated using the GATK Table Recalibration tool. Somatic single‐nucleotide variants and short indels were detected with the unmatched normal, using Mutect (1.1.6) and the SomaticIndelocator tool in GATK.^11^ Common and germline variants from the somatic variant candidates were filtered out with the common dbSNP (build 141; found in >1% of samples), Exome Aggregation Consortium (ExAC; r0.3.1, threshold frequency 0.001), Korean Reference Genome database (KRGDB), and an in‐house panel of normal variants. Final somatic variants were annotated using the Variant Effect Predictor (version 79) and then converted to the maf file format using vcf2maf (https://github.com/mskcc/vcf2maf). False‐positive variants were manually curated using the Integrative Genomics Viewer. For the analysis of structural variations, copy number variation (CNV) and rearrangement were evaluated using the CNVkit^12^ and BreaKmer^13^ algorithms, respectively. The GISTIC algorithm was applied to the segmented files (CNS) for the identification of significant focal and arm level amplifications and deletions.^14^ The GISTIC q‐value cutoff was set at 0.25 as per software instructions. Candidates for germline mutations or false positives for rearrangement alterations by BreaKmer were filtered out with an in‐house panel of normals and by manual review.

### 2.5 Statistical analysis

Data were descriptively summarized as frequency counts, medians, and ranges. A chi‐square or Fisher`s exact test was used for categorical variables and Mann–Whitney *U*‐test for continuous variables. The McNemar test was used to determine the agreement between the frequency of the NGS and conventional single‐targeting PCR tests. Overall survival (OS) was defined as the time from the initial date of systemic treatment to the date of death from any cause or to the date of the last follow‐up visit for alive patients. Survival rates were estimated using the Kaplan–Meier method, and comparisons were performed using the log‐rank test. The Cox proportional hazard regression model was used to evaluate prognostic factors for OS, and multivariate analysis included the potential prognostic factors that were significant (*p* < 0.1) in the univariate analysis. A two‐sided *p* value <0.05 was considered significant, and all statistical analyses were performed using IBM SPSS Statistics for Windows, Version 21.0 (IBM Corp.).

## 3 RESULTS

### 3.1 Clinical characteristics

Table 1 presents the baseline characteristics of the patients with lung adenocarcinoma who underwent NGS. The median age was 63 years (range, 25–86), and 58.8% of patients were male. Approximately 82% of patients had ECOG PS 0–1 and 43.7% were never smokers. According to the American Joint Committee on Cancer (AJCC) staging system 8th edition, 5.4% of patients were stage I, 3.6% were stage II, 6.4% were stage III, and 84.4% were stage IV. Among the 391 patients, 330 had initially metastatic or recurrent disease (stage IV) and were treated with systemic therapy. Each systemic targeted therapy for the actionable mutation type was administered to 173 patients (52.4%), regardless of the presence of actionable mutation and treatment lines. Of the 247 patients with metastatic or recurrent disease who had actionable mutations, targeted agents were administered to 159 patients (64.4%) based on the NGS reports, as follows: EGFR inhibitor (*n* = 101), ALK inhibitor (*n* = 34), RET inhibitor (*n* = 13), ROS‐1 inhibitor (*n* = 9), and BRAF inhibitor (*n* = 2). Eighty‐eight patients (35.6%) could not receive targeted therapy.

**TABLE 1:** Baseline characteristics of the patients

	All patients (*n* = 391, 100%)
Age (years) (median, range)	63 (25–86)
Sex	
Male	230 (58.8)
Female	161 (41.2)
ECOG PS	
0–1	324 (82.9)
2–4	30 (7.7)
Unknown	37 (9.5)
Smoking history	
Never smoker	171 (43.7)
Ex‐smoker	148 (37.9)
Current smoker	70 (17.9)
Unknown	2 (0.5)
Stage^*^	
I	21 (5.4)
II	14 (3.6)
III	25 (6.4)
IV	330 (84.4)
Prior therapy	
Surgery	143 (36.6)
Radiotherapy	30 (7.7)
Systemic treatment	330 (84.4)

Abbreviation: ECOG PS, Eastern Cooperative Oncology Group performance status.

* Stage according to the American Joint Committee on Cancer (AJCC) staging system 8^th^ edition in 390 patients with available data.

### 3.2 Mutation landscape

**FIGURE 1:**
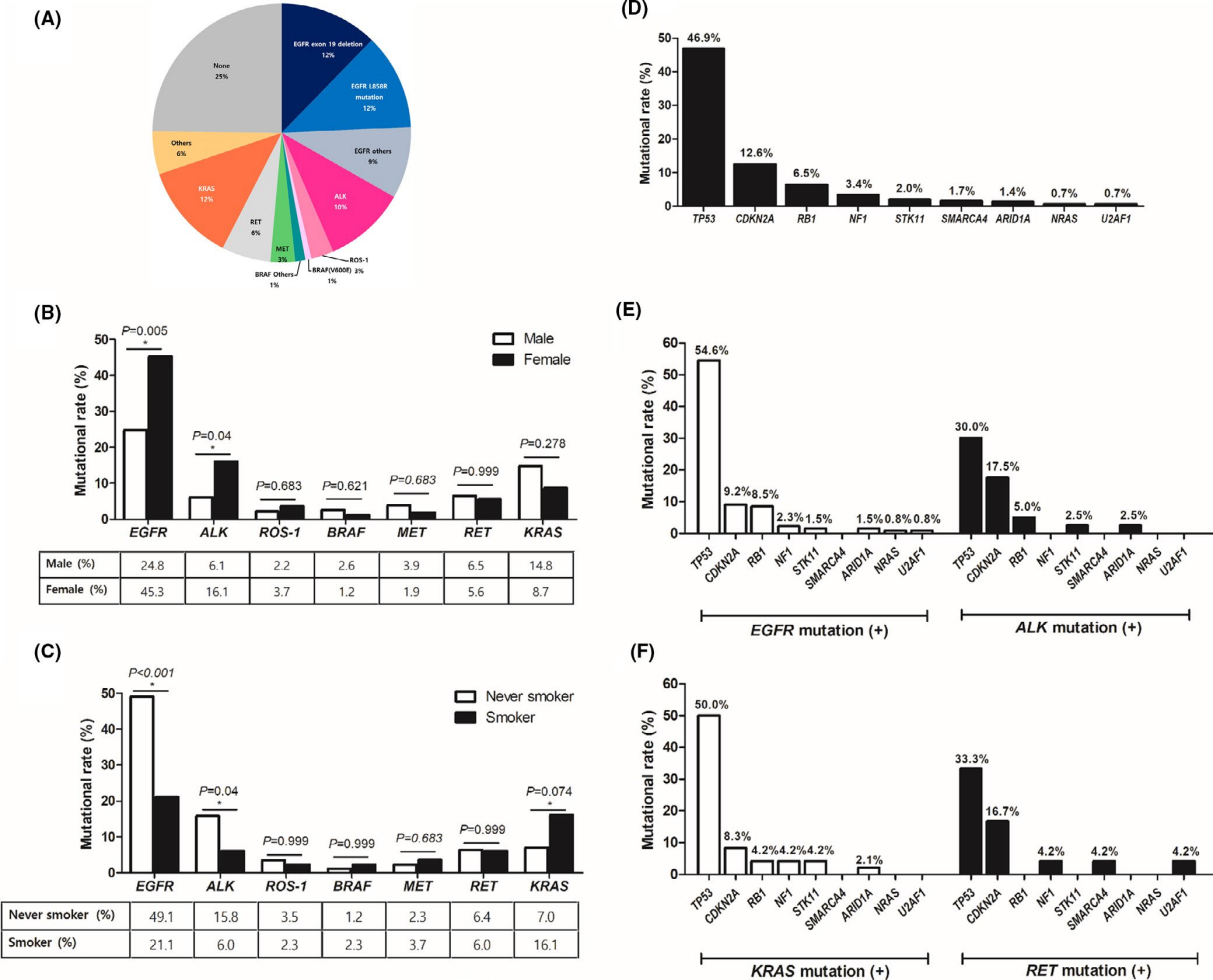
Frequency of (A) actionable genetic alterations according to (B) sex and (C) smoking history, and frequency of (D) co‐mutations, (E) co‐mutations with *EGFR* or *ALK* mutation, and (F) co‐mutations with *KRAS* or *RET* mutation in patients with lung adenocarcinoma. Others: *ERBB2*, *PIK3CA*, and *PTEN*

**FIGURE 2:**
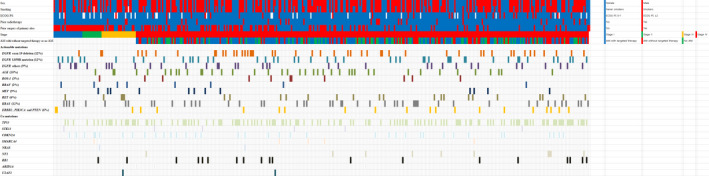
Genetic landscape of lung adenocarcinoma. Abbreviations: ECOG PS, Eastern Cooperative Oncology Group Performance Status; AM, actionable mutation

NGS was performed on available biopsy or surgical specimens prior to any line of treatments. Among 391 patients, at least one actionable genetic alteration was identified in 294 patients (75.2%), and 97 patients (24.8%) had no mutation. The most commonly mutated gene was *EGFR* (*n* = 130, 33.2%), involving *EGFR* exon 19 deletion (*n* = 48, 12.3%), L858R (*n* = 47, 12%), and others (*n* = 35, 9%), followed by *KRAS* (*n* = 48, 12.3%) and *ALK* (*n* = 40, 10.2%). The overall frequency of each mutation is presented in Figure 1A. *EGFR* and *ALK* mutations were more prevalent in females than in males (*EGFR*: 45.3% vs. 24.8%, *p* = 0.005; and *ALK*: 16.1% vs. 6.1%, *p* = 0.040) and more frequent in never smokers than in smokers (*EGFR*: 49.1% vs. 21.1%, *p* < 0.001; and *ALK*: 15.8% vs. 6.0%, *p* = 0.040) (Figure 1B,C). *KRAS* mutation was more frequent in smokers than in never smokers (*p* = 0.074) (Figure 1C). Among 294 patients with actionable mutations, 219 patients (74.5%) had co‐mutations. *TP53* was the most commonly mutated gene (46.9%), followed by *CDKN2A* (12.6%), *RB1* (6.5%), *NF1* (3.4%), *STK11* (2%), *SMARCA4* (1.7%), *ARID1A* (1.4%), *NRAS* (0.7%), and *U2AF1* (0.7%) (Figure 1D). The frequencies of the co‐mutations were similar between each actionable mutation group (Figure 1E,F). The genetic landscape and clinical characteristics are comprehensively shown in Figure 2.

Among 391 patients, conventional single‐targeting PCR for *EGFR* mutation was performed in 320 patients. The frequency of the *EGFR* mutation from NGS was similar to that of conventional PCR (33.8% vs. 27.8%) (*EGFR* exon 19 deletion; 13.1% vs. 12.2% and *EGFR* L858R mutation; 11.6% vs. 11.3%), while there were 25 discordant results, including 22 cases with *EGFR* mutation through NGS only (Table S2).

### 3.3 Actionable mutation and its impact on survival in patients treated with systemic therapy

**FIGURE 3:**
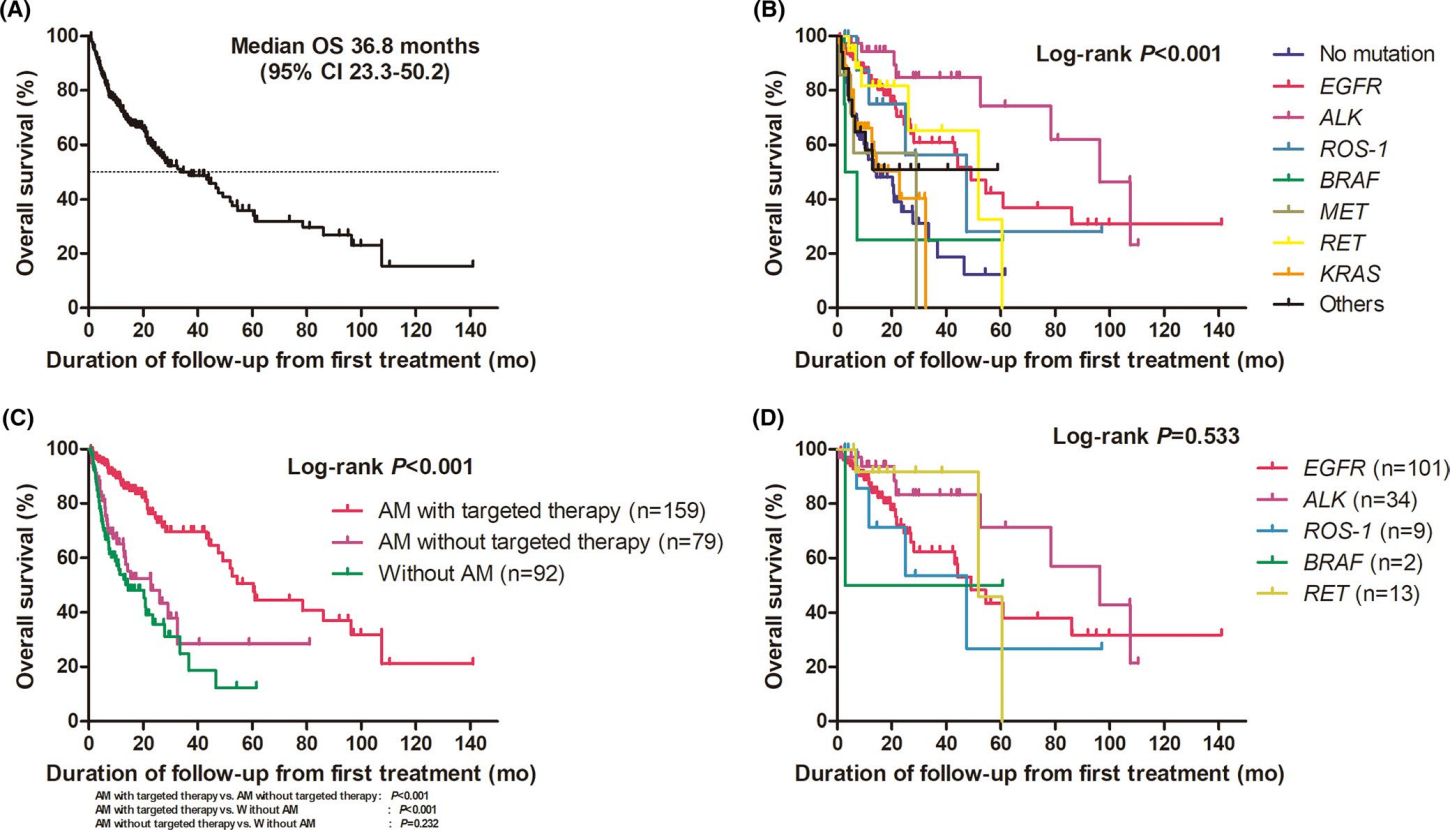
Kaplan–Meier curves for (A) overall survival in patients with stage IV disease who received systemic therapy, (B) overall survival according to actionable genetic alterations, (C) overall survival in patients with actionable genetic alterations according to the use of targeted therapy and patients without actionable genetic alterations, and (D) overall survival according to actionable genetic alterations in patients with actionable genetic alterations treated with targeted therapy. Abbreviations: AM, actionable mutation

Among the 391 patients, 330 were treated with systemic therapy. With a median follow‐up duration of 16.8 months (95% confidence interval [CI], 14.8–18.8), median OS was 36.8 months (95% CI, 23.3–50.2) (Figure 3A). Median OS according to mutation types is shown in Figure 3B and ranged from 5.1 (*BRAF*) to 96.4 months (*ALK*) (*p* < 0.001). Actionable mutation was identified in 247 patients (74.8%); 159 patients were treated with the corresponding targeted agents. The association of targeted agents and survival in patients with actionable mutations is shown in Figure 3C. Median OS in patients with actionable mutations treated with targeted therapy was significantly longer than that in patients with actionable mutations who could not receive targeted therapy (60.5 vs. 26.0 months, *p* < 0.001) (Figure 3C). There was no significant difference in median OS between patients with actionable mutations who could not receive targeted agents and patients without actionable mutations (26.0 vs. 14.3 months, *p* = 0.232) (Figure 3C). In patients with actionable mutations who were treated with targeted therapy, median OS according to mutation type was not significantly different (Figure 3D, *p* = 0.533), and median OS according to the presence of co‐mutations and number of co‐mutations was also not significantly different (Figure S1).

In the univariate analysis for OS, male, ECOG PS (≥2), smoking history, and no prior surgery of the primary site were significantly associated with poor OS, while actionable mutation with targeted therapy showed significant association with favorable OS (Table 2). In the multivariate analysis for OS, ECOG PS (≥2 vs. 0–1, hazard ratio [HR] 3.18, 95% CI 1.86–5.42; *p* < 0.001) and actionable mutation without or with targeted therapy (actionable mutation without targeted therapy vs. actionable mutation with targeted therapy, HR 2.58, 95% CI 1.57–4.25; *p* < 0.001) remained significant factors (Table 2).

**TABLE 2:** Univariate and multivariate analysis for overall survival in patients with stage IV disease

	Univariate analysis		Multivariate analysis	
	HR (95% CI)	*p*	HR (95% CI)	*p*
Age (>65 vs. ≤65 years)	1.14 (0.80–1.62)	0.476		
Sex (male vs. female)	1.97 (1.34–2.89)	0.001		
ECOG PS (≥2 vs. 0–1)	3.52 (2.11–5.86)	<0.001	3.18 (1.86–5.42)	<0.001
Smoking history (yes vs. no)	1.82 (1.25–2.64)	0.002		
Co‐mutation (yes vs. no)	1.02 (0.59–1.74)	0.957		
Prior radiotherapy (yes vs. no)	1.26 (0.71–2.25)	0.427		
Prior surgery of primary site (yes vs. no)	0.62 (0.40–0.95)	0.027		
Groups				
AM with targeted therapy	1		1	
AM without targeted therapy	2.85 (1.79–4.53)	<0.001	2.58 (1.57–4.25)	<0.001
Without AM	3.90 (2.53–5.99)	<0.001	3.84 (2.44–6.05)	<0.001

Abbreviations: AM, actionable mutation; CI, confidence interval; ECOG PS, Eastern Cooperative Oncology Group performance status; HR, hazard ratio.

## 4 DISCUSSION

This real‐world data showed the profile of actionable genetic alterations with comparable frequencies in lung adenocarcinoma by using NGS. Its clinical utility was also confirmed in clinical practice by improving survival in patients treated with the corresponding targeted therapy based on NGS reports. Three‐quarters of the patients had at least one genetic alteration, the rate and profiles of which were consistent with those of previous studies,^7^, ^8^, ^15^ and co‐mutations were also found in approximately three quarters of the patients with actionable mutations. *EGFR* and *KRAS* mutations were frequently identified in 33.2% and 12.3% of patients, respectively, and *ALK* (10.2%), *BRAF* (2%), *MET* (3%), *RET* (6%), *ROS*‐*1* (3%), *PIK3CA* or *PTEN* (1.3%), and *ERBB2* (3.6%) mutations were also identified. *TP53* (46.9%) and *CDKN2A* (12.6%) mutations were common co‐mutations in patients with actionable mutations. Among patients with actionable mutations, those treated with targeted therapy based on NGS reports lived significantly longer than those who did not receive such therapy (*p* < 0.001). Co‐mutations were not associated with survival following targeted therapy.

*EGFR* mutation is more prevalent in Asian patients, whereas *KRAS* mutation is more prevalent in Caucasian patients. A previous study identified *EGFR* and *KRAS* mutations in 21% and 25% of cases^15^ reported by the Lung Cancer Mutation Consortium, respectively, and 14% and 33% of cases in The Cancer Genome Atlas, respectively.^8^ In Asian patients, the rate of *EGFR* mutation was reported to be up to more than 50%, while *KRAS* mutation was found in less than 10%.^7^, ^16^, ^17^, ^18^ In accordance with these trends, *EGFR* mutation (33%) was the most common mutation, and *KRAS* mutation was found in only 12% in this study. However, the frequency of *EGFR* mutation was relatively lower than that in previous Asian studies. This may be due to underestimation in real practice circumstances. Although NGS is actively encouraged in real‐world practice since being included under the Korean national health insurance coverage, some patients with *EGFR* mutation initially identified by conventional single‐targeting PCR did not undergo NGS. The detection rate of a mutation can also be affected by differences in methodologies between studies. In addition, *ALK* mutation was similarly reported at a rate of less than 10%, and other genetic alterations in *BRAF*, *MET*, *RET*, and *ROS*‐*1* were similarly rare (<5%–6%) in Caucasian and Asian studies.

In this study, those with *EGFR* and *ALK* mutations were characterized as being female and non‐smokers, while *KRAS* mutation appeared to occur more frequently in smokers than in never smokers without statistical significance. Patients with *ALK* mutation were younger than those without such mutation (Mann‐Whitney *U*‐test, *p* = 0.002). These clinical features associated with *EGFR*, *ALK*, and *KRAS* mutations are well known in previous studies.^19^, ^20^, ^21^, ^22^, ^23^ Given reports describing that *KRAS* mutation is more common in male patients,^19^, ^20^, ^21^ this study found more *KRAS* mutations in males than in females, although this did not reach significance (14.8% vs. 8.7%, *p* = 0.278). Along with comparable frequencies, similar observations of these characteristics implied that conventional single‐target PCR could be substituted by NGS in actual clinical practice.

Through NGS, the identification of actionable genetic alterations can lead to the improvement of survival with targeted therapy in patients with lung cancer. The use of targeted agents in individual patients with *EGFR* and *ALK* mutations has been well‐documented.^2^, ^3^ In this study, median OS was significantly longer in patients with actionable mutations who were treated with targeted therapy than in patients who could not receive targeted therapy despite actionable mutations being identified (*p* < 0.001). In addition to *EGFR* and *ALK* mutations, the identification of additional *RET*, *ROS*‐*1*, and *BRAF* mutations and use of the corresponding targeted therapy improved survival in patients with actionable mutations. Recently, RET inhibitor (BLU‐667) has shown promising antitumor activity in terms of the response rate (60%) and disease control rate (91%) in patients with advanced *RET*‐mutated non‐small cell lung cancer (NSCLC).^24^ In a phase 2 trial, dabrafenib plus trametinib treatment showed a response rate of 64% in previously untreated patients with *BRAF*‐mutated NSCLC. Furthermore, *KRAS* mutation is a recently highlighted mutation,^25^ and KRAS inhibitor (AMG510) has shown antitumor activity in one case of partial response and two cases of stable disease among six patients with previously treated *KRAS*‐mutated lung adenocarcinoma.^26^ NGS is useful for identifying clinically meaningful genetic mutations, even rare ones, as well as common mutations, which can allow patients to receive better treatment targeting potential biomarkers.

There were some limitations to this retrospective study. First, given that NGS was not performed for all patients, specifically excluding some patients with *EGFR* mutations who were initially identified by conventional PCR, there could be a potential bias in investigating the mutation profile in lung adenocarcinoma. Second, as this was not a randomized study designed to compare survival in patients with actionable mutations according to targeted therapy, there could be differences in baseline characteristics and decision‐making depending on the treating physician's discretion. Improvement of survival with the corresponding targeted therapy should be carefully interpreted rather than definitive conclusions being made. Third, cancer‐specific survival could not be analyzed because of limited available data regarding causes of death. However, the strength of this study is that this real‐world data suggests clinical relevance of the widespread incorporation of NGS into routine clinical practice for treating lung adenocarcinoma.

## 5 CONCLUSION

This study revealed actionable genetic alterations in lung adenocarcinoma by using routine NGS and the improvement of survival with targeted therapy based on this sequencing method. Along with the strength of NGS in identifying various genetic alterations simultaneously, it can also be useful for providing guidance in real‐world practice when selecting treatment options for lung adenocarcinoma.

## CONFLICT OF INTEREST

The authors have no conflicts of interest to declare.

## Supporting information

Fig S1

## Data Availability

The datasets generated during and/or analyzed during the current study are available from the corresponding author (SWK) on reasonable request. The data are not publicly available due to them containing information that could compromise research participant privacy/consent.
