# Supplementary figures and images for: Real‐world utility of next‐generation sequencing for targeted gene analysis and its application to treatment in lung adenocarcinoma
Source: Cancer Med. 2021 May 7;10(10):3197–204. doi: 10.1002/cam4.3874 (PMC8124124; doi:10.1002/cam4.3874)

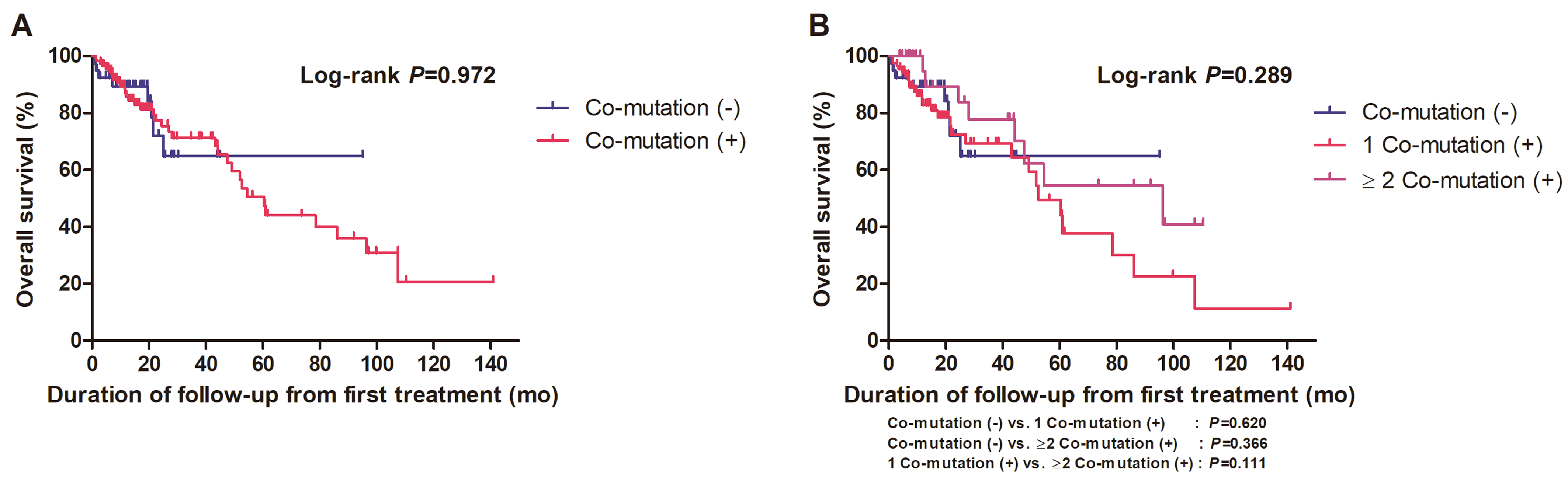

Supplement: Supplementary file 1 — Fig S1 [file CAM4-10-3197-s002.tif]
