# Supplementary material for: Real‐world utility of next‐generation sequencing for targeted gene analysis and its application to treatment in lung adenocarcinoma
Source: Cancer Med. 2021 May 7;10(10):3197–204. doi: 10.1002/cam4.3874 (PMC8124124; doi:10.1002/cam4.3874)
Supplement: Supplementary file 2 [file CAM4-10-3197-s001.docx]

**Table S1. Gene list of OncoPanel Version 3.0**

| **Entire exonic sequence for the detection of base substitutions, insertions/deletions, and copy number alterations** | **Partial intronic sequence for the detection of rearrangements** | **Hotspots for the detection of base substitutions and insertions/deletions** |
| --- | --- | --- |
| ABL1, ABL2, AKT1, AKT2, AKT3, ALK, APC, AR, ARAF, ARID1A, ARID1B, ARID2, ASXL1, ATM, ATR, ATRX, AURKA, AURKB, AURKC, AXIN1, AXL, BAP1, BARD1, BCL2, BRAF, BRCA1, BRCA2, BRD2, BRD3, BRD4, BRIP1, CBFB, CCND1, CCND2, CCND3, CCNE1, CD274, CDH1, CDK12, CDK4, CDK6, CDKN1A, CDKN1B, CDKN2A, CDKN2B, CDKN2C, CEBPA, CHEK2, CREBBP, CRKL, CSF1R, CTNNB1, DDR1, DDR2, DNMT3A, DOT1L, DPYD, EGFR, EPHA3, EPHB4, ERBB2, ERBB3, ERBB4, ERCC2, ERG, ERRFI1, ESR1, ETV1, ETV4, ETV5, ETV6, EWSR1, EZH2, FAM175A, FBXW7, FGFR1, FGFR2, FGFR3, FGFR4, FLCN, FLT1, FLT3, FLT4, FOXL2, GATA2, GEN1, GNA11, GNAQ, GNAS, HDAC9, HGF, HNF1A, HRAS, IDH1, IDH2, IGF1R, IGF2, JAK1, JAK2, JAK3, KDR, KIT, KMT2A, KRAS, LRP1B, MAP2K1, MAP2K2, MAP2K4, MAP3K1, MAP3K4, MAPK1, MAPK3, MAPK8, MCL1, MDM2, MDM4, MED12, MEN1, MET, MITF, MLH1, MPL, MRE11A, MSH2, MSH6, MTOR, MYC, MYCN, MYD88, NBN, NF1, NF2, NFKBIA, NKX2-1, NOTCH1, NOTCH2, NOTCH3, NOTCH4, NPM1, NRAS, NTRK1, NTRK2, NTRK3, NUTM1, PALB2, PBRM1, PDGFB, PDGFRA, PDGFRB, PIK3CA, PIK3CB, PIK3CD, PIK3R1, PIK3R2, PMS2, POLE, PPARG, PTCH1, PTCH2, PTEN, PTPN11, RAB35, RAD50, RAD51, RAD51C, RAD51D, RAF1, RARA, RB1, RET, RHEB, RICTOR, RNF43, ROS1, RSPO1, RSPO2, RUNX1, SMAD2, SMAD4, SMARCA4, SMARCB1, SMO, SPOP, SRC, STK11, SYK, TERT, TET2, TMPRSS2, TOP1, TOP2A, TP53, TSC1, TSC2, VHL, WT1, XPO1, XRCC2, ZNRF3 | ABL1, ALK, EGFR, EWSR1, NTRK1, RET, ROS1, TMPRSS2 | A1BG, ABCC5, ACVR1, ACVR2A, ADAMTS18, ADNP, AKAP7, ALX4, ANKRD20A3, ANTXR2, AP1S1, AP3S1, ARV1, ASH1L, ATP1A1, BAX, BCL7C, BLM, BTK, CALR, CAMSAP1L1, CASD1, CBL, CBWD3, CBX4, CBX5, CCDC73, CD3G, CD79B, CDH26, CEBPZ, CENPV, CEP290, CHERP, CISD2, CKAP2, CLEC18C, CLOCK, COBLL1, CPEB2, CRIPAK, CTGF, DDX11, DHX9, DLC1, DNAH12, DOCK3, DPAGT1, DYNC1I2, EBPL, EPPK1, FABP2, FAM115C, FAM153A, FAM18A, FAM193A, FAM75A6, FAM86C1, FAT1, FBXL3, FGFBP1, FMN2, FOXD4L6, FRG2B, FXR1, GOLGA8B, GOLGA8R, GRIN3B, GTPBP2, HIAT1, HIF1A, HS6ST1, HSPD1, IFITM1, IFITM3, IL10RB, IMPA1, INO80E, IRS1, KCTD16, KIAA1919, KLRC3, KNSTRN, KRT32, LIPT1, LMBRD1, MADCAM1, MAX, MBD3L3, MBD3L4, MTHFR, MUC12, MUC2, MVK, MYBL1, MYO1A, NBPF16, NBPF9, NDUFA6, NFE2L2, NGLY1, NIPA2, NNAT, NOMO1, NOS3, NOTCH2NL, NPEPPS, NUDT7, OR1D5, OR2T29, OR2T35, OR4M2, OR5B17, OR6B2, PABPC1, PCBP1, PCDHB16, PCDHB4, PCMTD1, PDXDC1, PMS2P11, POM121L3, PON2, PPP2R1A, PRAMEF11, PRAMEF13, PRAMEF19, PREX2, PRIM2, PSPC1, PTH2, PTPN11, PTPN3, RAC1, RASA4, RBBP8, RFX5, RGS12, RHOA, RNF222, RPN1, RRN3, RUFY2, SAA2, SAFB2, SCAF4, SEC63, SELRC1, SF3B1, SF3B5, SLC23A2, SPRR3, SSTR4, STAMBPL1, STAT3, STAT6, STAU2, SULT1A1, SULT6B1, SYCP1, SYNJ2, TAS2R19, TAS2R31, TBC1D3E, TCF7L2, TEAD2, TIMM23, TMEM14B, TMEM60, TMPRSS13, TNPO1, TPSAB1, TPSD1, U2AF1, UPK3BL, WDR55, WDR87, XAB2, ZBTB7C, ZFP37, ZNF141, ZNF198, ZNF518A, ZNF563 |

**Table S2. Frequency of *EGFR* mutation from next-generation sequencing (NGS) and conventional single-targeting PCR**

| 320 evaluable patients | *EGFR* mutation by NGS | No *EGFR* mutation by NGS |
| --- | --- | --- |
| *EGFR* mutation by conventional single-targeting PCR | 86 (26.9) | 3 (0.9) |
| No *EGFR* mutation by conventional single-targeting PCR | 22 (6.9)* | 209 (65.3) |

McNemar test, *P* < 0.001

*Among 22 patients, there were other sites of *EGFR* mutation (n = 14), *EGFR* exon 19 deletion (n = 6), and *EGFR* L858R mutation (n = 2).
